# Supplementary material for: Large Unstained Cells (LUC): A Novel Predictor of CDK4/6 Inhibitor Outcomes in HR+ HER2-Negative Metastatic Breast Cancer
Source: J Clin Med. 2024 Dec 31;14(1):173. doi: 10.3390/jcm14010173 (PMC11722146; doi:10.3390/jcm14010173)
Supplement: Supplementary file 1 [file jcm-14-00173-s001.zip › 10-Supplementary Table S2.pdf]

Supplementary Table S2: Patients and Tumor Characteristics According to the LUC Level				
		LUC < 0.11 (n=86)	LUC ≥ 0.11 (n=119)	*P* Value
Age, Median, IQR		58 (49-68)	55 (45-68)	
Gender		85 (99%)	117 (98%)	
Menopause	Pre	16 (19%)	27 (23%)	0.466
	Post	69 (80%)	90 (76%)	
	Male	1 (1%)	2 (1%)	
ECOG	0	2 (2%)	7 (6%)	0.896
	1	73 (87%)	99 (84%)	
	2	6 (7%)	10 (8%)	
	3	3 (4%)	2 (2%)	
HER2 Status	0	68 (80%)	88 (77%)	0.781
	1	11 (13%)	19 (16%)	
	2	6 (7%)	8 (7%)	
Metastatic Area	Bone	58 (67%)	77 (65%)	0.684
	Lung	6 (7%)	22 (19%)	0.018
	Liver	22 (26%)	26 (22%)	0.533
	Lymph Node	22 (26%)	26 (22%)	0.533
Received CT In Last Year		9 (11%)	15 (13%)	0.665
Denovo Metastatic		50 (58%)	80 (67%)	0.183
Line	First	72 (84%)	89 (75%)	0.124
	Second and later	14 (16%)	30 (25%)	
Received LHRH		8 (9%)	20 (17%)	0.123
Co- Administration ET	AI	63 (73%)	84 (71%)	0.676
	Fulvestrant	23 (27%)	35 (29%)	
CDK 4/6	Ribociclib	54 (63%)	80 (67%)	0.510
	Palbociclib	32 (37%)	39 (33%)	
Best Radiographic Response	CR	3 (4%)	12 (4%)	0.213
	PR	41 (53%)	57 (48%)	
	SD	26 (30%)	24 (20%)	
	PD	8 (9%)	10 (8%)	
	N/E	8 (9%)	16 (14%)	

Baseline patient and tumor characteristics stratified by LUC levels (< 0.11 vs. ≥ 0.11). The table includes comparisons of age, gender, menopausal status, ECOG performance status, HER2 status, metastatic sites, prior chemotherapy, and treatment regimens. Variables such as response to treatment and metastatic status (de novo vs. recurrent) are also presented. Data are expressed as median with interquartile range (IQR) or as counts/percentages. Abbreviations: LUC: large unstained cells; ECOG: Eastern Cooperative Oncology Group; AI: aromatase inhibitor; LHRH: luteinizing hormone-releasing hormone; CR: complete response; PR: partial response; SD: stable disease; PD: progressive disease; N/E: not evaluable.
