# Supplementary material for: Large Unstained Cells (LUC): A Novel Predictor of CDK4/6 Inhibitor Outcomes in HR+ HER2-Negative Metastatic Breast Cancer
Source: J Clin Med. 2024 Dec 31;14(1):173. doi: 10.3390/jcm14010173 (PMC11722146; doi:10.3390/jcm14010173)
Supplement: Supplementary file 1 [file jcm-14-00173-s001.zip › 9-Supplementary Table S1.pdf]

Supplementary Table S1: Previous studies on Large Unstained Cells (LUC)		
Author	Disease	Study Results
Merter et al.[19]	Autologous stem cell transplantation	An increase in LUC percentage correlates with CD34 stem cell count and predicts successful mobilization.
Fortes et al.[25]	Melanoma	No association was found between LUC/Lymphocyte ratio and mortality risk in melanoma.
Lanza et al. [22]	CLL	The number of LUC was correlated with the number of blasts in CLL and was found to be poor prognostic.
Hwang et al.[20]	Acute Leukemia	A correlation was found between leukemia relapse and high LUC levels.
Jerez et al.[21]	Plasma Cell Leukemia	A high LUC rate was detected in plasma cell leukemia.
Urbanowicz et al.[37]	Coronary Artery Disease	A correlation was found between the presence of coronary artery disease and high LUC levels in patients with aortic stenosis.
Eren et al.[38]	Diabetes Mellitus	LUC level and LUC percentage were found to be higher in patients than in non-diabetic patients.
Shin D et al.[23]	Varicella Infection	An increase in the percentage of LUC was observed during varicella infection and correlated with a decrease in the LUC level during clinical recovery.
Bastug et al.[39]	COVID Infection	The decrease in LUC percentage has a poor prognostic value.
Vanker et al.[16]	HIV Infection	The number of LUCs was found to be correlated with the number of CD38-expressing CD8 T lymphocytes.
Keseroglu et al.[40]	Epididymal-orchid	Higher LUC levels were detected in epididymal-orchid compared to testicular torsion.

This table summarizes key findings from studies investigating LUC across various diseases. It provides insights into the role of LUC in clinical and prognostic contexts.

LUC: Large Unstained Cells, CLL: Chronic Lymphocytic Leukemia, CD34: Cluster of Differentiation 34, a marker of hematopoietic stem cells, HIV: Human Immunodeficiency Virus, COVID: Coronavirus Disease, CD38: Cluster of Differentiation 38, a marker expressed on activated T lymphocytes
